# Evidence for Developmental Programming of Cerebral Laterality in Humans

**DOI:** 10.1371/journal.pone.0017071

**Published:** 2011-02-16

**Authors:** Alexander Jones, Clive Osmond, Keith M. Godfrey, David I. W. Phillips

**Affiliations:** 1 Centre for Cardiovascular Imaging, University College London Institute of Child Health, London, United Kingdom; 2 Lifecourse Epidemiology Unit, Medical Research Council, Southampton, United Kingdom; 3 Diet and Lifestyle Biomedical Research Unit, Southampton National Institute for Health Research Nutrition, University of Southampton, Southampton, United Kingdom; The University of Western Australia, Australia

## Abstract

Adverse fetal environments are associated with depression, reduced cognitive ability and increased stress responsiveness in later life, but underlying mechanisms are unknown. Environmental pressures on the fetus, resulting from variations in placental function and maternal nutrition, health and stress might alter neurodevelopment, promoting the development of some brain regions over others. As asymmetry of cerebral activity, with greater right hemisphere activity, has been associated with psychopathology, we hypothesized that regional specialization during fetal life might be reflected persistently in the relative activity of the cerebral hemispheres. We tested this hypothesis in 140 healthy 8–9 year-old children, using tympanic membrane temperature to assess relative blood flow to the cerebral hemispheres at rest and following psychosocial stress (Trier Social Stress Test for Children). Their birth weight and placental weight had already been measured when their mothers took part in a previous study of pregnancy outcomes. We found that children who had a smaller weight at birth had evidence of greater blood flow to the right hemisphere than to the left hemisphere (r = −.09, *P* = .29 at rest; r = −.18, *P* = .04 following stress). This finding was strengthened if the children had a relatively low birth weight for their placental weight (r = −.17, *P* = .05 at rest; r = −.31, *P* = .0005 following stress). Our findings suggest that lateralization of cerebral activity is influenced persistently by early developmental experiences, with possible consequences for long-term neurocognitive function.

## Introduction

Adverse fetal environments are associated with impaired neurocognitive function in later life, including depression [1], reduced cognitive performance [2] and increased stress responsiveness [3], [4], [5] but there has been little investigation of underlying processes. One hypothesis is that early neurodevelopment is affected by environmental pressures such as nutrient availability to the fetus, promoting the persistent dominance of some brain regions over others. At its most gross level, such regional specialization might be seen in the relative activity of the cerebral hemispheres.

It is well established that asymmetrical activation of the cerebral hemispheres is associated with altered affective states, including depression [6] and with heightened stress-responsiveness [7]. Neuroimaging in patients with unipolar depression shows hypometabolism of the left hemisphere (LH) and hypermetabolism of the right hemisphere (RH) [8], [9] and disease severity is correlated positively with RH hyperactivity [8]. Damage or dysfunction of the brain is associated with depression if the LH is affected, which promotes a shift towards greater relative RH activity, and with euphoria if the RH is affected, suggesting that functional balance of the hemispheres is important in determining mood [10], [11], [12], [13], [14], [15], [16]. Stimulation of the RH through exclusive presentation of emotionally aversive stimuli to the left visual field causes greater activation of stress regulatory systems (the hypothalamic pituitary adrenal axis (HPAA) and sympathetic nervous system) than does stimulation of the LH [7], [17], [18]. Functional magnetic resonance (MR) imaging (fMRI) has shown that the magnitude of HPAA and heart rate responses to stress are better correlated with blood flow in the RH than in the LH [19].

Technologies such as fMRI and positron emission tomography (PET) allow detailed study of cerebral activity but they have limitations that constrain their use in large epidemiological studies, particularly in studies of children. These include expense, limited access, noise or radiation exposure, motion intolerance, an environment that limits interaction with participants and that may stress them, and the requirement for time-consuming, technically challenging data analysis procedures. We sought out a technology that might give useful insights in such studies, enabling investigation of developmental effects on cerebral activity in cohort studies.

Tympanic membrane (TM) thermometry has emerged as a simple, inexpensive and non-invasive means to assess cerebral blood flow to the two hemispheres. Brain temperature and TM temperature (TMT) are usually higher than the temperature of incoming blood from the body. Thus, increased blood flow cools these tissues, leading to an inverse relationship between temperature and perfusion. Brain temperatures, estimated by MR thermometry, are related inversely to regional blood flow [20] and single photon emission computed tomography with the head in varied positions has shown that TMT relates inversely to ipsilateral cerebral perfusion, suggesting that blood flow to the TM and ipsilateral cerebral hemisphere have common vasomotor reflex control [21]. Cognitive tasks with asymmetrical cerebral activation cause decreased TMT on the side of the more active hemisphere compared to the opposite TMT [22], [23], [24], [25]. Thus, simultaneous bilateral TMT measurement may be used to assess cerebral activation and its laterality.

In this study, we evaluated cerebral activity and its laterality at rest and in response to a psychosocial stressor using TMT measurements to examine the hypothesis that regional specialization of the brain is affected persistently by differences in the fetal environment.

## Methods

We recruited 140 healthy children aged 8–9 years for a cross-sectional study. They had been followed since 12 weeks of gestation when their mothers took part in a study of children born in Southampton, United Kingdom [26]. At birth, they were weighed to the nearest five grams with calibrated digital scales. Their placenta was weighed similarly on calibrated digital scales after trimming by stripping the amnion to the cord, cutting the chorion at the edge of the placenta, and removing the cord flush with the placenta. Duration of gestation was estimated from menstrual history data confirmed by an early ultrasound scan. Childhood weight was measured with calibrated scales and subjects underwent the Trier Social Stress Test for Children [3], [27] with assessment of its effects on their TMT. The United Kingdom National Statistics Socio-economic Classification of the mother, her smoking habits during pregnancy and hand preference of the child were determined by parental questionnaire. All parents and children gave informed, written consent and the Southampton Local Research Ethics Committee approved the study.

### TSST-C

The children attended a clinical research facility for the TSST-C. The children were asked to stand in front of a video camera and microphone and perform an exciting story of their own invention, followed by a serial subtraction task for an audience of three adult strangers. They had 5 minutes to prepare before the stress test, which had a median duration of 11.1 (IQR 10.8–11.3) minutes. The original TSST-C protocol [27] was modified to reduce task difficulty appropriately for our younger age group, and we offered toys as potential rewards for high performance to increase motivation, with all children receiving a reward. In thanks for their participation, the children were also given a voucher for toys worth £10.

### TM thermometry

Simultaneous bilateral TMT was measured using a pair of infrared tympanic thermometers (Braun Thermoscan Pro 3000; Welch Allyn, Aston Abbotts, UK) on three occasions. After participants had acclimatized to their novel environment for one hour approximately, a baseline measure was taken, which preceded the TSST-C by 2 hours approximately. Further measures were taken at median delays of 4.5 (IQR 4–5.3) and 33 (IQR 32.4–33.9) minutes after completion of the TSST-C.

Each pair of measurements (left and right TMT) was taken twice, with the measurers and their thermometers swapping ears to allow correction for small differences in thermometer calibration and measurement technique. Side of first measurement and choice of thermometer for each user on each occasion were randomized by computer. Room temperature at the time of each measurement was recorded using a calibrated digital thermometer (RS 612–849, RS Components, Corby, UK). Thermometer calibration was regularly assessed using a Diatek 9600 blackbody device (error <0.2°C; Diatek Instruments, San Diego, USA) and no drift in measurement accuracy was found in the thermometers.

### Statistical methods

Mean left and mean right TMTs were calculated after swapping thermometers and their users to the opposite ears to minimize errors introduced by thermometer bias or measurement technique. TMT laterality (TMTL) was calculated by subtracting TMT on the right from that on the left. The polarity of this measure was chosen arbitrarily. Thus, TMTL should be greater if the RH is relatively more active than the LH, given the inverse relationship between TMT and activity. Average temperature of the two ears was used to assess overall cerebral activation, with lower temperatures indicating greater activity. Two-tailed student *t*-tests were used to compare temperatures between the sexes. Partial correlation was used to assess relationships between temperature measures and other variables. Because TMT was found to vary by sex and TMTL was found to relate to hand preference, adjustment was made for these factors in all analyses. Measures of fetal size were adjusted by linear regression with first and second order terms for gestational age to fit their slightly curvilinear relationship, yielding a small improvement in fit over linear adjustment. This ensured that our findings were not the result of variations in gestational length. Fetal growth in relation to growth of the placenta was assessed by considering the residual birth weight variation after that predicted by placental weight was removed by linear regression. This measure is uncorrelated with placental weight and might be preferred to the ratio of placental weight to birth weight because the ratio is correlated to both variables and is, therefore, more difficult to interpret. Analyses were repeated with current weight as a covariate to test the possibility that birth weight was acting as a proxy for current weight. Analyses were further repeated with maternal socioeconomic status and smoking included as covariates as these factors are known to influence birth weight and placental weight and could impact upon childhood neurodevelopment. For all statistical tests, *P*-values below .05 were taken to be significant.

## Results

**Figure 1:**
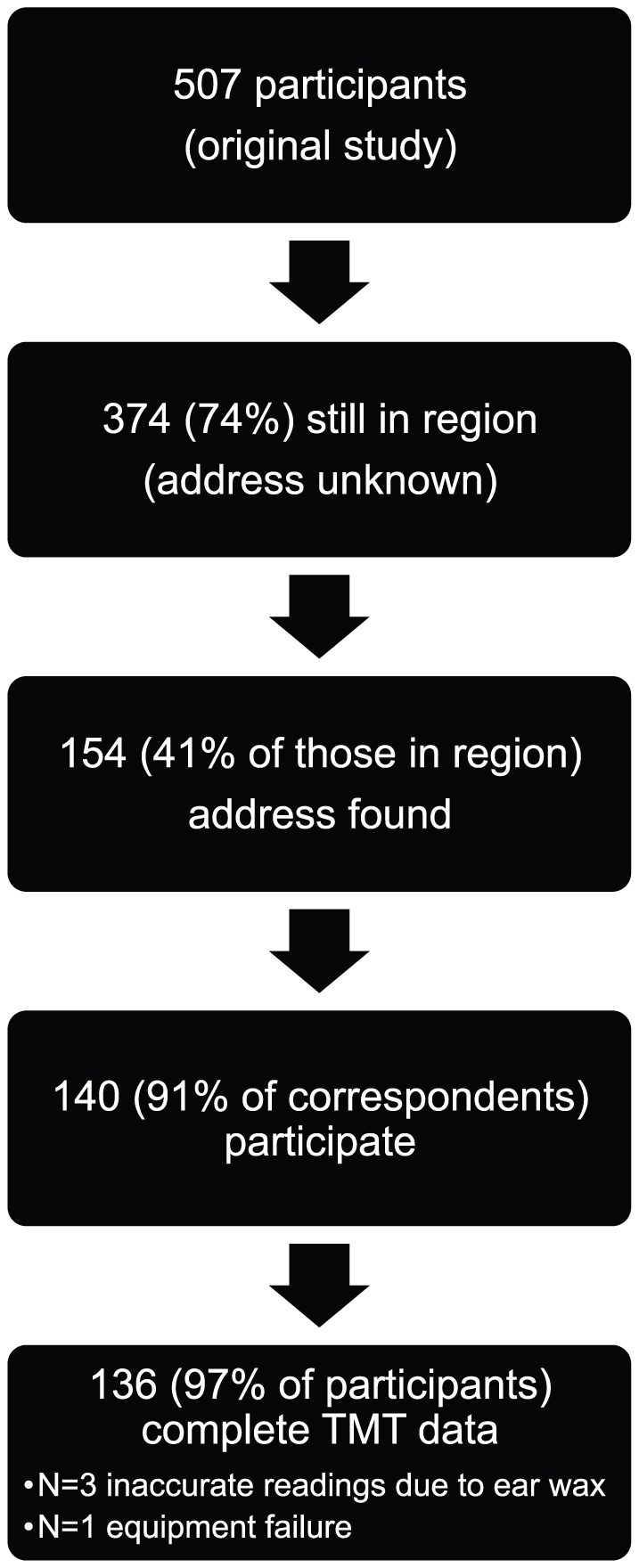
Flow chart of study participants.

Complete TMT measures were obtained in 136 of the participants (67 boys and 69 girls) with a median age of 8.9 (range 7.6–9.7) years (Figure 1). Their median gestational age was 40 (range 35–43) weeks. There were no significant differences in gestational age, birth weight or placental weight between participants and non-participating members of the original cohort. Mean baseline temperature was higher (*P* = .001) in girls (37.1°C, SD = 0.38) than boys (36.8°C, SD = 0.43). TMTL was distributed normally with a mean of 0.01°C (SD = 0.27°C, range −0.7°C to +1°C). There were no significant differences in TMTL according to sex. There was no significant relationship at any time of room temperature with either average TMT temperature or TMTL.

**Figure 2:**
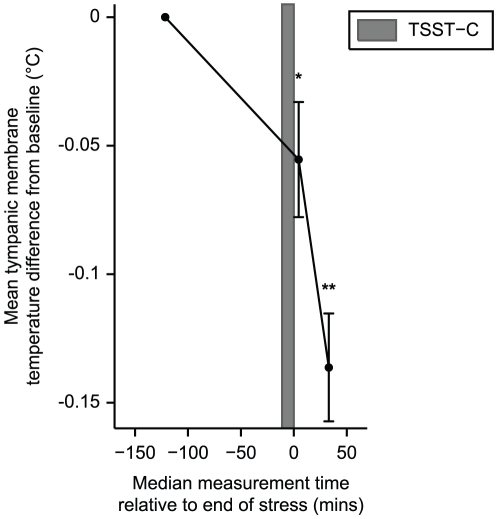
Mean (±SE) tympanic membrane temperature (average of left and right ear differences from baseline) falls following the Trier Social Stress Test for Children (TSST-C). ^*^ *P*<.05, ^**^ *P*<.0001. *P*-values refer to paired two-way student *t*-tests of measures following stress with those at baseline (120 minutes before stress).

Table 1 shows TMTs before and after the TSST-C. Mean Left and right TMTs fell by similar amounts in response to stress but TMTL did not change significantly. Because there was no significant difference between left and right falls in TMT, Figure 2 shows the average of left and right TMT differences from baseline pre-stress measures in response to the TSST-C.

**Table 1:** Mean (SD) tympanic membrane temperature for the left and right ears and the difference between them, prior to and following stress.

	Time (minutes) relative to end of stress		
	−120	+4.5	+33
Left ear	36.95 (0.42)	36.89 (0.46)*	36.81 (0.43)**
Right ear	36.94 (0.46)	36.89 (0.48)*	36.80 (0.45)**
Left – right difference	0.012 (0.33)	0.008 (0.34)	0.008 (0.32)

*P<.05, ^**^ *P*<.0001. *P*-values refer to paired two-way student *t*-tests of measures following stress with those at baseline (120 minutes before stress).

Mean TMTL was 0.2°C lower in left-handers than right-handers (*P* = .018) at baseline, but not significantly so following stress (0.13°C lower, *P* = .12). There was no significant difference, according to hand preference, in mean left or right TMT, considered in isolation, suggesting that neither hemisphere was more important in determining the relationship of hand preference with TMTL at baseline.

**Figure 3:**
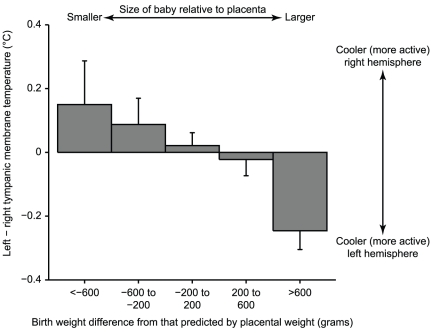
Mean (±SE) tympanic membrane temperature laterality 4.5 minutes after acute psychosocial stress relates to birth weight difference from that predicted by placental weight. *P* = .00048 for trend.

Table 2 shows the partial correlations of TMTL with measures of fetal and placental growth. Low birth weight was related inversely to TMTL and this was statistically significant 4.5 minutes after the TSST-C. This finding was markedly strengthened after birth weight was adjusted for placental weight, showing that small individuals at birth with relatively larger placentas have greater TMTL immediately following stress (Figure 3). There was a similar but weaker association at baseline and a trend (*P* = .06) towards such an association 33 minutes after stress. The findings did not differ significantly by sex and after adjustment for current weight, the findings were unaltered apart from the association of birth weight with TMTL 4.5 minutes after stress, which strengthened (r = −.23, *P* = .01) and the associations of birth weight adjusted for placental weight with TMTL at baseline, which strengthened (r = −.20, *P* = .03) and at 4.5 minutes after stress, which also strengthened (r = −.33, *P* = .0002). Thus, the findings with prenatal growth were independent of attained childhood weight.

**Table 2:** Partial correlations of measures of fetal and placental growth with tympanic membrane temperature laterality, before and after acute psychosocial stress.

	Time (minutes) relative to end of stress					
	−120	*P*	+4.5	*P*	+33	*P*
Birth weight	−.09	.29	−.18	.04	−.16	.07
Birth weight for placental weight*	−.17	.05	−.31	.0005	−.17	.06

*Birth weight adjusted for placental weight by linear regression. Correlations were adjusted for the participant's sex, hand preference and gestational age at birth.

To establish which hemisphere was most responsible for the associations between size at birth and TMTL shown in Table 2, a further set of secondary analyses were carried out relating size at birth to left and right TMTs individually (Table 3). There were minimal non-significant correlations with left TMT but both fetal growth measures were correlated positively and significantly with right TMT at baseline and 4.5 minutes after stress. This suggests that the associations of fetal growth parameters with TMTL are determined largely by variations in RH activity.

**Table 3:** Partial correlations of measures of fetal and placental growth with tympanic membrane temperature (TMT) in the left and right ears, before and after acute psychosocial stress.

	Time (minutes) relative to end of stress											
	Left TMT						Right TMT					
	−120	*P*	+4.5	*P*	+33	*P*	−120	*P*	+4.5	*P*	+33	*P*
Birth weight	.13	.14	.06	.51	.04	.69	.19	.03	.18	.04	.15	.09
Birth weight for placental weight*	.08	.40	−.01	.89	.01	.95	.20	.03	.20	.02	.13	.14

*Birth weight adjusted for placental weight by linear regression. Correlations were adjusted for the participant's sex, hand preference and gestational age at birth.

All fetal growth analyses were robust to further adjustment for maternal socioeconomic status and smoking during pregnancy.

## Discussion

To our knowledge, this study provides the first evidence that regional differences in cerebral activity may be influenced persistently by early developmental experiences. We found inverse linear associations between markers of fetal growth and RH activity 8–9 years later, which strengthened when fetal growth was considered in relation to placental growth. The associations seen at rest were markedly stronger in the period immediately following the TSST-C, suggesting that stress unmasks inherent differences in cerebral laterality. Thus, the activity of brain regions involved in the processing and response to stressful stimuli may be particularly susceptible to altered lateralization in fetal life. The associations were continuous across the normal ranges of birth weight and placental weight and not dependent on a threshold effect such as might be seen if they resulted only from medically significant intrauterine growth retardation. As the children were born at or near to term and birth size measures were adjusted for gestational age, the findings are not the result of prematurity.

We studied young children who are unlikely to have been affected by the psychological disorders or neurodegenerative processes that usually appear in later life. Our findings were robust to adjustment for current weight and maternal socioeconomic status and smoking, increasing the likelihood that they represent the direct effect of altered neurodevelopment.

We found that hand preference was related to TMT differences indicative of greater baseline activity in the hemisphere that was not dominant for motor functions. While this finding may seem counter-intuitive, it agrees with long-established evidence that overall hemispheric activity is usually greater in the hemisphere that is not dominant for motor activities [28]. However, the reasons for this remain a matter of speculation. Because of this finding, further analyses of TMTL were adjusted for hand preference.

We used birth weight as a summary measure of fetal growth. As the heritable component of birth weight is small [29], it may be considered a non-specific reflection of adverse intrauterine influences, to which fetal growth is highly sensitive [30]. As these influences may be disparate, including variations in placental function and maternal nutrition, health and stress, our findings point to a need for future investigations to establish which particular developmental influences may alter later laterality of cerebral activity.

In healthy pregnancies, fetal and placental weights generally increase proportionately. In childhood, we found that birth weight adjusted for placental weight was a stronger determinant of increased RH activation following stress than birth weight alone. This suggests that disproportionate growth of the fetus and placenta is more important than fetal growth restriction alone in determining cerebral laterality. In our data, a relatively small baby with a relatively large placenta was associated with a neurodevelopmental outcome that might be associated with impaired neurocognitive function in later life. This is not the first time that such disproportion has been linked to later pathology. For example, increased placenta to fetus weight ratio has been linked to risk of hypertension in adulthood [31], [32]. Interestingly, however, so has a decreased placenta to fetus weight ratio [32], [33] and other components of the metabolic syndrome also appear to have a ‘U’-shaped relationship with placenta to fetus weight ratio [34]. Thus, although fetal growth in disproportion to that of the placenta is likely to indicate a departure from normal, healthy growth at some stage during pregnancy, underlying causes may differ. Therefore, this indicator of fetal adversity, like birth weight, remains non-specific but may be more sensitive, given its stronger associations with asymmetry of cerebral activity in our study.

In our study, a relatively small fetus with a relatively large placenta might be considered detrimental. Although a precise pathogenesis cannot be determined, existing data allows for some speculation on the type of placental adaptation that may have occurred. Maternal undernutrition during pregnancy may stimulate placental overgrowth as an adaptation to poor nutrient supply in an effort to improve fetal nutrition in later gestation [34]. In early gestation, the placenta is less developed and has the greatest growth potential. Therefore, adaptation to poor nutrient supply might be best achieved through placental hypertrophy. In later gestation, placental enlargement may be limited by maternal constraint and diminished developmental plasticity and adaptations to improve nutrient transfer usually take the form of altered placental structure and function, rather than size. Thus, the gestational timing of adverse influences on the pregnancy, such as maternal malnutrition, may be a determinant of the direction of feto-placental growth disproportion. There is animal [35], [36], [37] and human [38] data that supports this, with undernutrition in early pregnancy being associated generally with increased placental weight relative to that of the fetus and there is some evidence that undernutrition in late pregnancy can be associated with a decreased placenta to fetus weight ratio.

Recently, maternal nutritional status has been implicated as a moderating factor that might determine the polarity of feto-placental growth disproportion [39]. In the offspring of short, low socioeconomic status mothers, a relatively small placenta was associated with later hypertension, whilst in the offspring of tall, wealthier mothers, a relatively large placenta was associated with hypertension. This was interpreted as evidence that compensatory placental enlargement is more likely in mothers who are more likely to have good nutrition in later pregnancy. As none of the parents in our study fell into the ‘routine work’ or ‘never worked/long-term unemployed’ categories at the lower end of the socioeconomic scale, this may explain why only feto-placental disproportion with a relatively large placenta was related to an adverse outcome in our study and no ‘U’-shaped relationship was found.

There is evidence that prenatal influences other than maternal nutrition may play an important role. In a recent large-scale study of the influence of maternal stress on human pregnancy outcomes [40], increased maternal stress levels were associated with placental enlargement. Although the extent to which maternal diet was altered by stress was not examined, it is more likely that maternal stress mediators rather than nutrient availability altered placental growth. This raises the interesting possibility that the findings in the present study could be the result of combined effects of maternal stress mediators on both placental growth and the development of brain regions involved in offspring stress responses. However, this is speculative and requires further investigation.

In vertebrates, neuroanatomical asymmetries appear very early in gestation as the result of complex expression of genetic signaling pathways [41], [42]. Of particular interest is that many of the genes involved in these pathways are susceptible to epigenetic regulation [43], [44] and, thus, to the influence of external environmental regulation. Thus, future studies might investigate epigenetic programming of these genes as a possible explanation for our findings.

Our study has a number of limitations. Our markers of fetal developmental experiences are non-specific and further studies should address specific influences on neurodevelopment in both early and late gestation. Our measure of cerebral activation is indirect, is likely to have limited sensitivity and cannot specify which regions of the brain are involved. Future work with fMRI or PET has the potential to address these issues in selected groups of subjects. Finally, due to the disruptive nature of our measurement technique, we were unable to measure TMT during the TSST-C. Thus, our first measure was several minutes after the termination of stress. It is unknown how rapidly changes in cerebral activation are reflected in TMT changes and it is therefore possible that our findings represent a reduced ability to return RH activation to baseline following acute stress rather than differences in acute RH response to stress. However, the presence of similar albeit weaker associations at rest argues against this. Further work with TMT measurement could explore continuous measures to fully assess the response to stress.

We have provided evidence that early development of the fetus influences the lateralization of cerebral activity, particularly following stress, in early childhood. Risk of depression and enhanced stress responsivity, with their consequent risks for ill health including cardiovascular disease, is increased in people who were small at birth. Our findings suggest that persistent differences in cerebral lateralization determined during gestation offer a potential explanation for this. Brain regions involved in the processing and response to stressful stimuli may be particularly susceptible to these effects.
